# Pleuropulmonary Involvement in Patients with Systemic Lupus Erythematosus as Detected by High-Resolution CT Scans: Clinical and Immunological Association

**DOI:** 10.3390/medicina61020181

**Published:** 2025-01-22

**Authors:** Manal A. Hasan, Maram A. Alismail, Danah R. Bokhari, Rehab F. Alghamdi, Zainab E. Alhalal, Safi G. Alqatari, Mohammed D. Al Shubbar

**Affiliations:** 1 Division of Rheumatology, Department of Internal Medicine, King Fahad Hospital of the University, Imam Abdulrahman Bin Faisal University, Dammam 34221, Saudi Arabia; mahasan@iau.edu.sa (M.A.H.); sagqatari@iau.edu.sa (S.G.A.); 2 College of Medicine, Imam Abdulrahman Bin Faisal University, Dammam 34221, Saudi Arabia; maramalismail@gmail.com (M.A.A.); bokhari175@gmail.com (D.R.B.); dr.rehab.f.alghamdi@gmail.com (R.F.A.); zaynabalhalal@gmail.com (Z.E.A.)

**Keywords:** systemic lupus erythematosus, pleuropulmonary involvement, pulmonary manifestations, high-resolution computed tomography, risk factors

## Abstract

*Background*: Systemic lupus erythematosus (SLE) is a complex autoimmune disease that leads to systemic inflammation and damage across various organs, including the respiratory system. The prevalence of pleuropulmonary manifestations in SLE patients, particularly in Saudi Arabia, remains underexplored. *Objective*: This study aims to determine the frequency of pleuropulmonary involvement in SLE patients at King Fahd Hospital of the University (KFUH), Khobar, Saudi Arabia, and identify factors associated with the presence of such findings. *Method*: A retrospective analysis was conducted on adult SLE patients at KFUH, using hospital medical records for data collection on demographic characteristics, clinical features, and immunological markers. Pleuropulmonary involvement was defined based on high-resolution computed tomography (HRCT) findings. Statistical analyses evaluated associations between pleuropulmonary findings and clinical or immunological parameters. *Results*: Out of 207 SLE patients (mean age 39.9 years, 92.8% female), 17.4% showed pleuropulmonary involvement on HRCT, with pleurisy being the most prevalent manifestation, followed by pulmonary nodules. Significant associations were found between pleuropulmonary involvement and positive rheumatoid factor (*p* = 0.002), cardiac involvement (*p* = 0.002), and higher SLICC/ACR damage index scores (*p* = 0.001). Patients with positive rheumatoid factor and elevated SLICC/ACR damage index had increased odds of developing pleuropulmonary complications (3.73- and 7.28-fold, respectively). *Conclusions*: Pleuropulmonary involvement in SLE patients is associated with clinical and immunological markers, including rheumatoid factor, cardiac involvement, and higher SLICC/ACR damage index scores. Recognizing these associations may improve the early detection and targeted management of high-risk patients.

## 1. Introduction

Systemic lupus erythematosus (SLE) is a complex autoimmune disease characterized by systemic inflammation, which can affect multiple organs, including the respiratory system [1]. Pulmonary involvement in SLE is diverse, ranging from pleuritis and pulmonary hypertension to interstitial lung disease and pulmonary embolism. Despite their frequency and potential severity, the reported prevalence of pulmonary manifestations in SLE patients varies widely across studies, with estimates ranging from 20% to 90% [2]. In the Middle East, the reported rate of pulmonary involvement varied from 4.9% to 33%, with pleural effusion being the most common pulmonary disease reported [3]. However, a comprehensive understanding of pulmonary involvement patterns and predictors in SLE remains limited. Several studies have explored the risk factors and predictors associated with pleuropulmonary involvement in SLE patients. Clinical factors such as higher disease activity scores, increased damage indices, and involvement of organs like the kidneys and heart have been suggested to elevate the risk of pulmonary complications [4,5,6]. Immunological factors, including the presence of specific autoantibodies like antiphospholipid antibodies, anti-ribonucleoprotein (anti-RNP), and rheumatoid factor, have also been implicated in increasing the likelihood of pleuropulmonary manifestations [7,8,9]. Despite these associations, findings across studies have been inconsistent, and the exact relationships between these factors and pulmonary involvement in SLE are not fully understood. This uncertainty underscores the need for further research to clarify these associations, which could enhance early detection and inform targeted interventions for high-risk patients.

This study aims to address the gap in understanding pleuropulmonary involvement in SLE patients in Saudi Arabia by examining a cohort from King Fahd Hospital of the University (KFUH) in Khobar. Utilizing high-resolution computed tomography (HRCT) scans, we seek to accurately determine the frequency of pleuropulmonary manifestations in these patients. Furthermore, this study investigates clinical and immunological factors associated with pleuropulmonary involvement, including demographic characteristics, immunological markers, and SLE-related damage indices. By identifying specific patterns and predictors of pulmonary complications, we aim to enhance understanding of SLE’s respiratory manifestations in this population. This knowledge could support early detection efforts and inform targeted interventions for high-risk patients, ultimately contributing to refined screening practices and improved management strategies.

## 2. Materials and Methods

### 2.1. Subjects

A retrospective study was conducted at the Rheumatology Department of King Fahad University Hospital (KFUH) in Al-Khobar, Saudi Arabia. The study sample consisted of SLE patients of both genders, aged 18 years and above, and diagnosed with SLE based on the American College of Rheumatology (ACR), Systemic Lupus International Collaborating Clinics (SLICC), or European League Against Rheumatism (EULAR)/ACR classification criteria [10,11,12]. Patients diagnosed with a coexistent connective tissue disease, such as rheumatoid arthritis, systemic sclerosis, Sjogren’s syndrome, or mixed connective tissue diseases, were excluded.

### 2.2. Materials and Procedures

This study adhered to the Strengthening the Reporting of Observational Studies in Epidemiology (STROBE) guidelines to ensure transparent and comprehensive reporting of observational research. The STROBE checklist, which outlines the essential items to include in observational studies, is provided as Supplementary Table S1.

The electronic health records of 207 patients who followed up at lupus clinics in KFUH from 1 January 2011 to 31 December 2020 (for a total of 10 years) were collected. The data included the sociodemographic, clinical, laboratory, serological, radiological, and histological profiles of all patients.

In addition to the sociodemographic characteristics (sex, age, nationality, ethnicity, and smoking status), the clinical data of SLE were gathered from patients’ history notes. These data included mucocutaneous involvement (defined as oral/nasopharyngeal ulcers, alopecia, a facial rash, or discoid lupus), musculoskeletal involvement (defined as arthralgia, arthritis, synovitis, tendinitis, myositis, or avascular necrosis), neuropsychiatric involvement (defined as psychosis, headache, seizures, and demyelinating diseases), and renal involvement based on 24 h urine protein, creatinine, and/or a positive kidney biopsy. Cardiac involvement was detected by echocardiography, and pleuropulmonary involvement was based on a positive radiological profile as diagnosed by HRCT scans of the chest. Comorbidities, including diabetes mellitus (DM), hypertension, and dyslipidemia, were also documented. The Systemic Lupus International Collaborating Clinics/American College of Rheumatology (SLICC/ACR) damage index, developed and validated to measure damage in 12 different systems, was used and calculated for each patient [13].

The laboratory data were obtained to check for the hematological involvement of the disease. Positive hematological findings were considered by measuring the complete blood counts for anemia (defined as hemoglobin <11.5 g/dL in female patients and <13 g/dL in male patients), leukopenia (defined as white blood cell count <4000/μL), and thrombocytopenia (defined as platelets <100,000/μL) [14]. The presence of renal involvement was established by abnormal renal function tests (Cr > 1.3 mg/d), persistent proteinuria (3.5 g/24 h), or confirmation of lupus nephritis via kidney biopsy utilizing ACR, SLICC, or EULAR/ACR criteria.

Immunological markers, such as antinuclear antibodies (ANAs), were defined as positive if the titer was ≥1:80, as assessed through indirect immunofluorescence (IIF). Moreover, the presence of positive anti-double-stranded DNA (anti-dsDNA), anti-Smith (anti-Sm) antibodies, antiribonucleoprotein (anti-RNP), and rheumatoid factor (RF) was confirmed based on the local laboratory’s reference range for serological parameters. Additional markers, such as lupus anticoagulants, anticardiolipin antibodies, anti–anti-B2-glycoproteins, anti-Ro antibodies, and anti-La antibodies, were considered positive according to the ACR, SLICC, EULAR/ACR, and ICS classification criteria. Serum levels of complements, specifically C3 and C4 (≤90 and ≤13 mg/dL, respectively, based on the local laboratory’s reference range), were also recorded. Positive results for the erythrocyte sedimentation rate (ESR) and C-reactive protein test (CRP) were interpreted per the local laboratory’s reference range to indicate elevated inflammatory markers.

Patients’ imaging profiles were utilized to identify cardiac and pleuropulmonary involvement through positive radiological observations on echocardiograms, chest X-rays, and high-resolution computed tomography (HRCT). The detection of cardiovascular involvement was documented based on echocardiographic results, encompassing valvular, endocardial, myocardial, and/or pericardial involvement. Valvular involvement was defined by the presence of valve thickening, vegetation, and/or malfunction, whereas myocardial involvement denoted the presence of myocardial hypertrophy, remodeling, or systolic dysfunction. The presence of pericardial effusion was perceived as pericardial involvement [15].

Pleuropulmonary involvement was documented based on patients’ clinical presentations in correlation to their radiological findings of SLE-related damage to the lung parenchyma, vessels, airways, and pleura. Lung parenchymal involvement was documented based on HRCT findings that suggested interstitial lung disease, lupus pneumonitis, pulmonary nodule, and/or atelectasis. Interstitial lung disease usually showed diffuse or bibasilar infiltrate or a honeycombing pattern in HRCT, while lupus pneumonitis was suggested by a diffuse acinar filling pattern in the lower lobes and commonly coexisted with pleural effusion. Similarly, pulmonary nodules were determined based on the finding of homogenous, well-defined nodules with normal surrounding parenchyma. Vascular involvements included positive findings for pulmonary arterial hypertension, pulmonary embolism (PE), lung infarction, and pulmonary hemorrhage [16]. Pulmonary arterial hypertension was defined as a mean pulmonary artery pressure (PAP) equal to or greater than 25 mmHg at rest or 30 mmHg with exercise in echocardiogram reports [16]. Pulmonary CT angiography was used to confirm the PE diagnosis. Additionally, peripheral wedge-shaped pulmonary consolidations suggesting pulmonary infarcts were recorded. The presence of radiographic diffuse alveolar opacities served as a hallmark sign for identifying patients with a pulmonary hemorrhage. Airway involvement, such as bronchiectasis, was also determined through HRCT imaging. Finally, pleuritis represented the primary pleural involvement of SLE patients in this study sample.

Coexisting comorbidities, such as hypertension, were defined as systolic BP ≥ 130 mmHg and/or diastolic BP ≥ 80 on at least three occasions during clinical visits [17]. Diabetes mellitus (DM) was defined as hemoglobin A1C ≥ 6.5%, fasting blood glucose ≥ 126 mg/dL, or random blood glucose ≥ 200 mg/dl with symptoms [18]. Dyslipidemia was defined as total cholesterol of 200 mg/dL, triglycerides of 150 mg/dL, low-density lipoprotein cholesterol of 100 mg/dL, or high-density lipoprotein cholesterol less than 40 mg/dL in male patients and 50 mg/dL in female patients [19].

Coexisting comorbidities, such as hypertension, were defined as systolic BP ≥ 130 mmHg and/or diastolic BP ≥ 80 on at least three occasions during clinical visits [17]. Diabetes mellitus (DM) was defined as hemoglobin A1C ≥ 6.5%, fasting blood glucose ≥ 126 mg/dL, or random blood glucose ≥ 200 mg/dl with symptoms [18]. Dyslipidemia was defined as total cholesterol of 200 mg/dL, triglycerides of 150 mg/dL, low-density lipoprotein cholesterol of 100 mg/dL, or high-density lipoprotein cholesterol less than 40 mg/dL in male patients and 50 mg/dL in female patients [19].

### 2.3. Statistical Analysis

The data were analyzed using Statistical Packages for Social Sciences (SPSS) version 26 (IBM Corp.: Armonk, NY, USA). Categorical variables were elaborated as numbers and percentages. Continuous variables were shown as mean and standard deviation. The association between pleuropulmonary involvement and the basic demographic characteristics, immunological, and hematological markers was performed using the Fischer exact test and the independent sample *t*-test. Significant results were then gathered in a multiple binary logistic regression model to determine the significant independent predictors of pleuropulmonary involvement. The cutoff point to determine statistical significance was set at *p* < 0.05. There were no missing data in this study. All collected variables were complete and included in the analysis.

## 3. Results

This study involved 207 patients. Table 1 shows that the average age of the patients was 39.9 years, and most were female (92.8%). Nearly all (93.2%) belonged to the Arab ethnic group, and the majority (91.3%) were Saudi citizens. Not a single patient admitted to smoking.

Positive ANA (100%), positive anti-ds-DNA (89.8%), and low complement levels (73.9%), in that order, were the most prevalent immunological markers. The least prevalent antibody found was anti-Jo1 (1.9%). The study population had high rates of anemia (88.5%) and increased ESR (84.5%) (Table 2).

**Figure 1:**
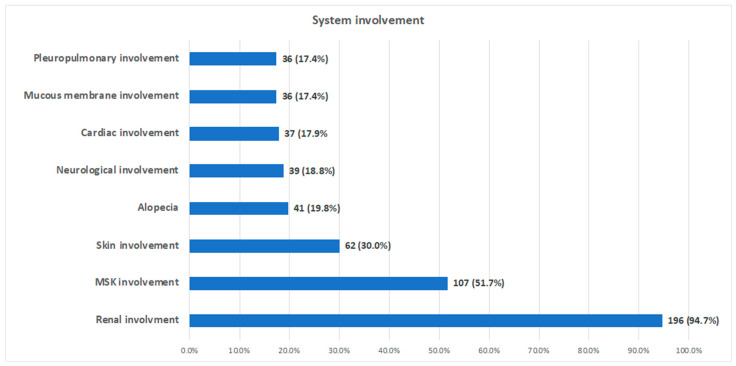
System involvement in the study population.

Renal involvement was the most prevalent, accounting for 91.3% of the participants’ high urine protein and 57% of their elevated serum creatinine levels. The biopsy results verified lupus nephritis in 26.1% of the study population. Thirty-six individuals (17%) had pleuropulmonary involvement (Figure 1). Furthermore, half of the patients’ SLICC/ACR damage index scores were high. That is, 103 patients had a score of 0–1, and 104 patients had a score of higher than one.

As shown in Table 3, pleuritis accounted for 36% of all pleuropulmonary involvements, with pulmonary nodules and bronchiectasis following closely behind. There were no cases of shrinking lung syndromes.

When measuring the association between pleuropulmonary involvement and patient’s socio-demographic, immunological, and clinical factors (Table 4), it was observed that patients with pleuropulmonary changes on HRCT had a significantly higher prevalence of positive rheumatoid factor (*p* = 0.002), cardiac involvement (*p* = 0.002), and a higher SLICC/ACR damage index (*p* = 0.001).

A multiple binary logistic regression analysis was performed to predict the independent risk factor for pleuropulmonary involvement in SLE patients. Among the variables examined, independent risk factors associated with pleuropulmonary involvement included positive rheumatoid factor (OR: 3.733, 95% CI: 1.143–12.188, *p* = 0.029), positive anti-β2 glycoprotein 1 IgG (OR: 5.326, 95% CI: 1.334–21.264, *p* = 0.018), and having SICC/ACR Damage >1 (OR: 7.284, 95% CI: 2.083–25.466, *p* = 0.002) (Table 5).

## 4. Discussion

In this retrospective cross-sectional study of 207 patients with SLE at King Fahd Hospital of the University in Khobar, Saudi Arabia, we investigated the frequency of pleuropulmonary involvement detected via HRCT and identified associated clinical and immunological risk factors. Our findings add to the growing body of evidence surrounding pleuropulmonary complications in SLE, emphasizing their clinical relevance and the necessity for early detection and intervention. In particular, we identified several factors that were strongly associated with an increased risk of pleuropulmonary involvement, including positive rheumatoid factor, cardiac involvement, higher SLICC/ACR damage index scores, and positive antiphospholipid antibodies. In this discussion, we compare our results with those from regional and international studies and explore the clinical implications of our findings.

### 4.1. Frequency of Pleuropulmonary Involvement

Pleuropulmonary involvement in SLE shows significant variability across populations, influenced by factors such as diagnostic criteria, imaging modalities, and patient demographics. In our study, pleuropulmonary manifestations were identified in 17.4% of patients, aligning with certain international studies yet lower than other reports from the region [3,6]. This discrepancy may stem from our focus on HRCT findings over clinical symptoms or other imaging techniques, such as X-ray or ultrasound, which could result in underestimating transient or acute pulmonary events. Nonetheless, HRCT remains a superior tool for identifying subclinical or early lung involvement, which is critical for the timely management of SLE-related pulmonary complications.

Pleuritis and pleural effusion rank among the most prevalent pulmonary manifestations in SLE, affecting 30–50% of patients over the course of the disease [2]. In our cohort, pleuritis emerged as the most frequent finding, observed in 36.1% of patients, aligning with prior research. On an international scale, the GLADEL cohort in Latin America reported pleuritis in 24% of patients, identifying it as the most prevalent pleuropulmonary manifestation [5]. Likewise, a study from Spain documented pleural disease in 21% of patients [4]. In contrast, national data from Saudi Arabia show a notably higher prevalence, with pleural effusion observed in 49% and pleuritis in 29% of patients in a study by Alamoudi et al. [3]. Conversely, other local studies, such as that by Alhammadi et al., report contrasting figures, with pleuritis found in only 6.1% and pleural effusion in 4% of patients [7]. The differences between local studies remain unclear but may reflect variations in diagnostic methodology and patient characteristics, such as disease activity or duration [4,6,20]. These factors could significantly impact the detection and reporting of pleuropulmonary manifestations in SLE patients.

Pulmonary nodules, though infrequently reported in SLE patients, were the second most common finding in our cohort, observed in 27.8% of those with pulmonary involvement. This aligns with the 26% reported in a study from Egypt, likely attributable to the use of HRCT [6]. However, the clinical significance of these findings remains uncertain, and any association with poorer patient outcomes is unclear. In contrast, pulmonary hypertension is a well-established, serious, and potentially life-threatening complication in SLE, often secondary to recurrent pulmonary emboli, ILD, or cardiac involvement [2]. Pulmonary hypertension was observed in 5.6% of our patients with pleuropulmonary involvement, a rate consistent with international reports [4,5]. Nationally, the prevalence of PH in SLE patients in Saudi Arabia varies significantly, ranging from 1.9% [21] to a much higher 23% [3]. A recent meta-analysis calculated a pooled prevalence of 8%, with variations likely reflecting differences in diagnostic methods and patient populations [22].

ILD was identified in 19.4% of patients, similar to the 16% reported in Turkey [23], but higher than the 4% observed in Saudi Arabia [7]. Pulmonary embolism was present in 5.6% of our patients, a rate between the 7% and 3.6% reported in two Saudi studies [3,7]. Though rare, pulmonary hemorrhage occurred in 8.3% of patients, a rate significantly higher than in other reports [4,7]. Additionally, atelectasis was observed in 22.2% and pneumonitis in 11.1% of patients, both comparable to findings from Saudi Arabia [3]. Notably, no cases of shrinking lung syndrome were detected in our cohort, consistent with its low prevalence rates of 0.8% and 1.6% reported in Spain and Latin America, respectively [4,5].

### 4.2. Risk Factors and Predictors of Pleuropulmonary Involvement

Cardiac involvement emerged as a significant independent predictor of pleuropulmonary manifestations in SLE patients in our study. This finding aligns with previous research indicating that both ischemic and non-ischemic heart diseases are independently linked to pleuropulmonary complications, including pleurisy, pulmonary hypertension, and pneumonitis [4,5]. The current literature lacks a clear explanation for the specific relationship between cardiac and pulmonary involvement in SLE. While some overlap may be secondary to the shared physiological pathways between the heart and lungs, a unique connection between cardiac dysfunction and pleuropulmonary manifestations in SLE remains unestablished. This relationship could largely be an effect of the physiological interdependence between these systems rather than a direct pathological link specific to SLE. For example, pulmonary hypertension may develop as a secondary consequence of cardiomyopathy or ventricular dysfunction, illustrating how cardiac issues can indirectly impact pulmonary function through their physiological interdependence [2,24]. Although other organ involvement was not associated with pleuropulmonary complications in our study, previous research has identified certain organ manifestations that show a strong association with pleuropulmonary involvement. These manifestations include mucocutaneous involvement, the Raynaud phenomenon, and renal involvement [4,5,7,25,26]. Additionally, our study identified anemia as the most prevalent hematological finding in SLE patients, followed by leukopenia and thrombocytopenia (Table 5). Alomair et al. reported similar findings, with rates of anemia (60%), leukopenia (37%), and thrombocytopenia (15%) [21]. However, no significant association was found between these hematological abnormalities and pulmonary manifestations.

In contrast, the association between APS and pleuropulmonary involvement in SLE is well-documented and has a strong pathophysiological basis [5,8,27,28,29,30]. In our study, specific antiphospholipid antibodies were linked to pulmonary manifestations; notably, IgG anticardiolipin antibodies showed marginal significance in their association with pulmonary involvement. Meanwhile, logistic regression identified IgG glycoprotein antibodies as a statistically significant predictive factor for these manifestations, underscoring their role in pleuropulmonary risk. These findings align with previous research, including a meta-analysis, which highlighted IgG anticardiolipin antibodies as being specifically associated with increased risk of PAH and pleuropulmonary complications, without similar findings for other antibodies [28]. Additional cohort studies support these associations [5,27].

Positive RF emerged as a significant independent predictor of pleuropulmonary involvement in our study. This association is supported by previous research, which shows that RF positivity correlates with an increased risk of pulmonary hypertension in SLE patients [9,26]. The presence of RF in SLE may indicate a subset of patients with heightened immune activation, which could predispose them to an increased risk of both pulmonary and vascular complications. This subset of RF-positive SLE patients may experience more severe disease manifestations, including pleuropulmonary involvement and potential vascular compromise. Other antibodies potentially associated with pulmonary complications in SLE were not found in our cohort. For instance, anti-RNP antibodies have been linked to a shorter time to pulmonary damage onset [20], and an association with pulmonary complications has also been observed in patients with anti-RNP positivity [4]. However, it remains unclear whether anti-RNP positivity alone drives these complications or if it indicates a subset of patients with mixed connective tissue disease.

In Saudi Arabia, additional antibodies, including anti-CCP and low complement levels, have been associated with pleuropulmonary complications [3]. Although we observed no correlation with low complement levels in our cohort, consistent with other studies [31], anti-CCP was not assessed, as it is rarely positive in SLE and may instead suggest an overlap syndrome. Further research into the role of anti-CCP in pleuropulmonary complications in SLE could help clarify its potential impact.

Our study also found that a higher SLICC/ACR damage index was significantly associated with pleuropulmonary involvement in SLE, consistent with findings from other studies. For example, a Spanish cohort reported elevated SLEDAI scores and SLICC/ACR damage index scores in SLE patients with respiratory complications, highlighting the link between disease activity and pulmonary involvement [4]. Another study found that patients with pulmonary manifestations had significantly higher SLEDAI-2k and SLICC/ACR scores, which correlated with pulmonary abnormalities on HRCT and ultrasound, including ground-glass opacities, honeycombing, interlobular septal thickening, pleural thickening, and effusion [6]. Similarly, higher disease activity at the time of diagnosis has been associated with a shorter time to pulmonary damage onset [20]. Together, these findings suggest that severe disease activity and accumulated organ damage increase the risk of pleuropulmonary complications in SLE.

### 4.3. Clinical Implications

Our study’s findings have significant implications for SLE management, particularly in the early detection, monitoring, and treatment of pleuropulmonary complications. Key risk factors—positive RF, cardiac involvement, elevated SLICC/ACR damage index scores, and antiphospholipid antibody positivity—underscore the need for tailored interventions and close monitoring to improve patient outcomes.

Given that pleuropulmonary manifestations in SLE patients range from 17.4% in our study to over 50% in others [6], early detection is essential. HRCT is valuable for detecting subclinical pulmonary involvement, as Hamed et al. found abnormalities in 52.63% of patients [6]. Routine HRCT screening, especially for patients with positive RF, aPL antibodies, or high disease activity, could allow clinicians to detect pulmonary complications early and initiate timely management to prevent irreversible damage.

Risk stratification is essential for optimizing SLE patient care. Identifying patients with positive RF, cardiac involvement, elevated damage scores, or aPL positivity enables personalized treatment and monitoring. For example, patients with positive RF, cardiac involvement, and elevated SLICC/ACR damage index scores may benefit from more comprehensive monitoring and early intervention. These patients are at higher risk for pulmonary complications, including pulmonary arterial hypertension (PAH), interstitial lung disease (ILD), and pleural effusions. A tailored approach might include regular HRCT scans for subclinical lung involvement, echocardiography for PAH monitoring, and pulmonary function tests to track respiratory decline.

We hypothesize that immune dysregulation involving multiple autoantibodies, such as RF and glycoprotein IgG antibodies, contributes to the increased risk of pulmonary complications. Further research into these immunological mechanisms may reveal therapeutic targets and improve risk stratification in SLE. Clinically, our study supports routine assessment of RF and aPL antibodies, along with regular HRCT screening, to facilitate early detection of pulmonary involvement and prevent progression to irreversible damage.

### 4.4. Strengths and Limitations

A key strength of our study is the use of HRCT, which enables the detection of subtle pulmonary abnormalities that conventional radiography may miss. This enhances our ability to detect early pleuropulmonary involvement in SLE patients. Furthermore, the comprehensive collection of clinical, immunological, and serological data allowed for a thorough analysis of risk factors, offering valuable insights into their association with pulmonary complications. However, several limitations should be acknowledged. Our single-center, retrospective design may limit the generalizability of findings to other populations and healthcare settings. Selection bias may also be present, as conducting the study in a tertiary care center likely attracted more severe or complicated SLE cases, potentially leading to an overestimation of pleuropulmonary involvement. Another consideration in this study is the potential overlap introduced by the pulmonary component of the SLICC/ACR damage index, which includes pulmonary-specific items reflecting long-term sequelae of SLE-related lung involvement. This overlap may influence the observed association between higher SLICC/ACR damage index scores and pleuropulmonary involvement, potentially amplifying the relationship. While the SLICC/ACR damage index remains a validated and widely used tool for assessing cumulative organ damage in SLE, its inclusion of organ-specific components necessitates cautious interpretation of the findings. Future studies could explore alternative approaches, such as adjusting the damage index to exclude specific organ items, to further validate and refine these associations while maintaining the integrity of cumulative damage assessments. Another limitation is the potential underestimation of subclinical cases. Although HRCT is highly sensitive, some subclinical or transient pulmonary manifestations might have been missed if not captured within the study’s time frame, particularly in patients without frequent imaging. Additionally, cultural factors may have influenced smoking status reporting, as none of the patients admitted smoking, potentially impacting the assessment of smoking as a risk factor for pulmonary complications.

## 5. Conclusions

In summary, our study highlights the frequency of pleuropulmonary involvement in SLE patients and identifies strong associations with key clinical and immunological factors, such as positive rheumatoid factor, cardiac involvement, and elevated SLICC/ACR damage index scores. While our findings align with some previous research, variations in prevalence across studies underscore the need for further investigation to refine screening and management strategies for SLE-related pulmonary complications.

## Figures and Tables

**Table 1:** Socio-demographic characteristics of the study population (*n* = 207).

Sociodemographic Variable	N (%)
Age in years (mean ± SD)	39.9 ± 11.6
Gender	
Male	15 (07.2%)
Female	192 (92.8%)
Ethnicity	
Arab	203 (98%)
Asian	04 (2%)
Smoking status	
Smoker	0
Non-smoker	207 (100%)

**Table 2:** Serological and hematological parameters in the study population (*n* = 207).

Variables	N (%)
Immunological markers	
Positive ANA	207 (100%)
Positive Anti-ds-DNA	185 (89.8%)
Low C3 or C4	153 (73.9%)
Positive Tests for Antiphospholipid Antibodies	98 (47.3%)
Positive Anti-Smith	84 (40.6%)
Positive Anti-Ro60 (Anti-SSA)	74 (35.7%)
Positive Anti-La (Anti-SSB)	27 (13.0%)
Positive Anti-RNP (Antiribonucleoprotein)	65 (31.4%)
Positive Anti-CENP-B (Anti-Centromere)	40 (19.3%)
Positive Rheumatoid Factor	38 (18.4%)
Positive Anti-Scl70	07 (03.4%)
Positive Anti-Jo1	04 (01.9%)
Hematological markers	
Anemia	183 (88.4%)
Leukopenia	143 (69.1%)
Thrombocytopenia	53 (25.6%)
Inflammatory Markers	
ESR > 20 mm/h	175 (84.5%)
CRP > 0.3 mg/dL	155 (74.9%)

Values are the number (percentage). Anti-ds-DNA = anti-double-stranded deoxyribonucleic acid antibodies; C3 = complement 3; C4 = complement 4; anti-Ro (anti-SSA) = anti–Sjögren’s syndrome-related antigen A autoantibodies; anti-La (anti-SSB) = anti–Sjögren’s syndrome-related antigen B autoantibodies; anti-RNP = anti-ribonucleoprotein antibodies; anti-CENP-B = anti-centromere proteins B antibodies; anti-Scl70 = anti-topoisomerase I; anti-Jo1 = anti-histidyl tRNA synthetase antibodies; ESR = erythrocyte sedimentation rate; CRP = C-reactive protein test.

**Table 3:** Patterns of pleuropulmonary involvement in HRCT-chest (*n* = 36).

Pattern of Pleuropulmonary Involvement	N (%)
Pleuritis	13 (36.1%)
Pulmonary Nodule	10 (27.8%)
Bronchiectasis	09 (25.0%)
Atelectasis	08 (22.2%)
Pulmonary Fibrosis/ILD	07 (19.4%)
Pneumonitis	04 (11.1%)
Pulmonary Hemorrhage	03 (8.3%)
Pulmonary Arterial Hypertension	02 (5.6%)
Pulmonary Embolism	02 (5.6%)
Lung Infarction	01 (2.8%)
Shrinking Lung Syndrome	0 (0.00%)

Values are the number (percentage). ILD = interstitial lung disease.

**Table 4:** Variables associated with the presence of pleuropulmonary changes on HRCT-chest in patients with SLE (*n* = 207).

Factor	Pleuropulmonary Changes on HRCT Chest		*p*-Value ^§^
	Yes N (%)	No N (%)	
Age group in years (mean ± SD)	43.2 ± 13.4	39.2 ± 11.1	0.056 ^‡^
Gender			
Male	04 (11.1%)	11 (6.4%)	0.325
Female	32 (88.9%)	160 (93.6%)	
Immunological markers			
Positive ANA	36 (100%)	171 (100%)	1.000
Positive Anti-Smith	14 (38.9%)	70 (40.9%)	0.820
Positive Anti-ds-DNA	33 (91.7%)	152 (88.9%)	0.623
Positive Anti-Ro60 (Anti-SSA)	15 (41.7%)	59 (34.5%)	0.415
Positive Anti-La (Anti-SSB)	07 (19.4%)	20 (11.7%)	0.210
Positive Anti-RNP (Antiribonucleoprotein)	12 (33.3%)	53 (31.0%)	0.783
Positive Anti-Jo1	01 (02.8%)	03 (01.8%)	0.685
Positive Anti-Scl70	02 (5.6%)	05 (02.9%)	0.427
Positive Anti-CENP-B (Anti-Centromere)	07 (19.4%)	33 (19.3%)	0.984
Low C3	25 (69.4%)	114 (66.7%)	0.747
Low C4	21 (58.3%)	104 (60.8%)	0.782
Low C3 or C4	25 (69.4%)	128 (74.9%)	0.502
Positive Rheumatoid Factor	13 (36.1%)	25 (14.6%)	0.002 **
Positive Anticardiolipin (IgM)	3 (8.3%)	20 (11.7%)	0.560
Positive Anticardiolipin (IgG)	6 (16.7%)	55 (32.2%)	0.064
Positive Anti-β2 Glycoprotein 1 (IgM)	3 (8.3%)	15 (8.8%)	0.932
Positive Anti-β2 Glycoprotein 1 (IgG)	9 (25%)	42 (24.6%)	0.956
Lupus Anticoagulant	6 (16.7%)	39 (22.8%)	0.417
Positive Anticardiolipin/Anticardiolipin/Glycoprotein (IgM)/Glycoprotein(IgM)/Lupus Anticoagulant	14 (38.9%)	84 (49.1%)	0.264
Hematology markers			
Anemia	31 (86.1%)	152 (88.9%)	0.636
Leukopenia	25 (69.4%)	118 (69.0%)	0.959
Thrombocytopenia	9 (25.0%)	44 (25.7%)	0.927
Anemia/Leukopenia/Thrombocytopenia	33 (91.7%)	155 (90.6%)	0.847
Inflammatory markers			
ESR > 20 mm/h	29 (80.6%)	146 (85.4%)	0.467
CRP > 0.3 mg/dL	25 (69.4%)	130 (76.0%)	0.408
ESR > 20 mm/h or CRP > 0.3	30 (83.3%)	151 (88.3%)	0.413
System involvement			
Mucous Membrane Involvement	07 (19.4%)	29 (17.0%)	0.721
Skin Involvement	10 (27.8%)	52 (30.4%)	0.754
Alopecia	10 (27.8%)	31 (18.1%)	0.187
MSK Involvement	19 (52.8%)	88 (51.5%)	0.886
Neurological Involvement	05 (13.9%)	34 (19.9%)	0.403
Chronic Disease (DM, HTN, or DLP)	14 (38.9%)	44 (25.7%)	0.110
Renal Involvement	33 (91.7%)	163 (95.3%)	0.374
Cardiac Involvement	13 (36.1%)	24 (14.0%)	0.002 **
SLICC/ACR Damage			
0–1	09 (25.0%)	94 (55.0%)	0.001 **
>1	27 (75.0%)	77 (45.0%)	

Values are the number (percentage). HRCT = high-resolution computed tomography; anti-ds-DNA = anti-double-stranded deoxyribonucleic acid antibodies; anti-Ro (anti-SSA) = anti-Sjögren’s syndrome-related antigen A autoantibodies; anti-La (anti-SSB) = anti-Sjögren’s syndrome-related antigen B autoantibodies; anti-RNP = anti-ribonucleoprotein antibodies; anti-Scl70 = anti-topoisomerase I; anti-Jo1 = anti-histidyl tRNA aynthetase antibodies; anti-CENP-B = anti-centromere proteins B antibodies; C3 = complement 3; C4 = complement 4; ESR = erythrocyte sedimentation rate; CRP = C-reactive protein test; MSK involvement = musculoskeletal involvement; DM = diabetes mellitus; HTN = hypertension; DLP = dyslipidemia; SLICC/ACR = Systemic Lupus International Collaborating Clinics/American College of Rheumatology. ^§^ *p*-value has been calculated using the Fischer exact test. ^‡^ *p*-value has been calculated using an independent sample *t*-test. ** Significant at *p* < 0.05 level.

**Table 5:** Risk factors predicting HRCT changes in patients with SLE.

Factors	AOR	95% CI	*p*-Value
Gender	1.142	0.219–5.950	0.874
Positive Anti-Smith	0.817	0.277–2.410	0.714
Positive Anti-DNA	1.251	0.211–7.415	0.805
Positive Anti-Ro60 (Anti-SSA)	1.226	0.401–3.752	0.721
Positive Anti-La (Anti-SSB)	1.125	0.262–4.819	0.874
Positive Anti-RNP (Antiribonucleoprotein)	1.109	0.359–3.423	0.857
Positive Anti-Jo1	5.155	0.150–177.115	0.363
Positive Anti-Scl70	2.609	0.182–37.395	0.480
Positive Anti-CENP-B (Anti-Centromere)	1.263	0.363–4.396	0.713
Low C3 or C4	0.573	0.182–1.808	0.342
Positive rheumatoid factor	3.733	1.143–12.188	0.029
Positive Anticardiolipin IgM	0.119	0.011–1.287	0.080
Positive Anticardiolipin (IgG)	0.223	0.050–1.000	0.050
Positive Anti-β2 Glycoprotein 1 (IgM)	1.579	0.202–12.375	0.663
Positive Anti-β2 Glycoprotein 1 (IgG)	5.326	1.334–21.264	0.018
Lupus Anticoagulant	1.136	0.317–4.068	0.845
Anemia	1.032	0.208–5.119	0.969
Leukopenia	1.338	0.407–4.396	0.631
Thrombocytopenia	0.812	0.245–2.699	0.735
ESR > 20 mm/h	1.486	0.303–7.287	0.625
CRP > 0.3 mg/dL	0.474	0.125–1.801	0.273
Mucous Membrane Involvement	1.858	0.544–6.352	0.323
Skin Involvement	0.817	0.242–2.750	0.744
Alopecia	0.493	0.121–2.003	0.322
MSK Involvement	1.347	0.475–3.820	0.576
Neurological Involvement	0.229	0.062–0.842	0.127
Chronic Disease (DM, HTN, or DLP)	1.514	0.566–4.052	0.409
Renal Involvement	0.166	0.019–1.467	0.106
Cardiac Involvement	2.487	0.779–7.940	0.124
SICC/ACR Damage >1	7.284	2.083–25.466	0.002

anti-Ro (anti-SSA) = anti-Sjögren’s syndrome-related antigen A autoantibodies; anti-La (anti-SSB) = anti-Sjögren’s syndrome-related antigen B autoantibodies; anti-RNP = anti-ribonucleoprotein antibodies; anti-Scl70 = anti-topoisomerase I; anti-Jo1 = anti-histidyl tRNA synthetase antibodies; anti-CENP-B = anti-centromere proteins B antibodies; C3 = complement 3; C4 = complement 4; ESR = *erythrocyte sedimentation rate*; CRP = C-reactive protein test; MSK involvement = *musculoskeletal* involvement; DM = diabetes mellitus; HTN = hypertension; DLP = dyslipidemia; CI—confidence interval.

## Data Availability

The data used in this study are not publicly available due to privacy concerns and institutional regulations. However, de-identified data supporting the findings of this study may be available from the corresponding author upon reasonable request, provided it complies with institutional data sharing policies and privacy requirements.

## Supplementary Materials

The following supporting information can be downloaded at: https://www.mdpi.com/article/10.3390/medicina61020181/s1, Table S1: STROBE Statement
